# Medical teachers' affective domain teaching dilemma and path exploration: a cross-sectional study

**DOI:** 10.1186/s12909-022-03870-1

**Published:** 2022-12-20

**Authors:** Ziyan Zhang, Qiongyin Hu, Chunyan Xu, Jianmin Zhou, Junhong Li

**Affiliations:** 1 School of Innovation and Entrepreneurship, Wenzhou Medical University, Chashan Campus, Zhongxin North Road, Ouhai District, Wenzhou City, Zhejiang Province China; 2 Department of Cardiology, Lishui Central Hospital and the Fifth Affiliated Hospital of Wenzhou Medical University, Lishui City, Zhejiang Province China; 3 School of Pharmaceutical Sciences, Wenzhou Medical University, Zhongxin North Road, Ouhai District, Wenzhou City, Zhejiang Province China; 4 School of Public Health and Management, Wenzhou Medical University, Wenzhou City, Zhejiang Province China

**Keywords:** Affective domain, Cross-sectional, Experiential learning, Medical education, Medical teacher, Survey

## Abstract

**Background:**

In the student-centered modern teaching environment of higher education, the affective domain was of great value to the overall development of medical students and the sustainable development of medical education. However, in the teaching practice of the medical specialty in Our country, there are still a lot of phenomena that pay attention to knowledge teaching but neglect affective education. Compared with affective domain, teachers tended to focus on the learning of specialized knowledge and skills. This paper investigated the attitudes and evaluations of teachers and students of medical school on affective education, analyzed the current situation and problems of teaching in the affective educauion of medical professions, and explored the path of combining the affective field and medical profession from the perspective of medical teachers.

**Methods:**

A questionnaire survey was conducted among medical teachers and students across the country. Using the self-appointed teacher scale and the student scale to obtain their ratings of all dimensions of teaching in the affective field through a free online tool called "WenJuanXing". Descriptive statistics and multiple regression analysis methods were used to analyze the data to explore the obstacles in affective education and the factors affecting the outcome of affective education.

**Results:**

A total of 523 medical teachers and 3268 medical students were surveyed, according to the results of questionnaire data analysis, there are differences in the needs of senior and lower grade students in various dimensions of affective education, and the current Chinese medical teachers carrying out affective education are facing unclear positioning and interpretation of affective education goals, lack of affective experience in teaching methods, lack of affective education evaluation norms, lack of continuity and progressivity of affective cultivation, and school organizational mechanisms need to be improved.

**Conclusions:**

This paper had some suggestions aimed at the above problem. Firstly, It is necessary to strengthen the construction of the organizational mechanism of medical universities, provide them with guarantees and training according to the characteristics of teachers of different teaching ages, and comprehensively improve teachers' affective literacy from the inside out. Secondly, teachers should design clear progressive goals and content systems of affective education, constructing an evaluation system of affective education in the experiential teaching method.

## Background

Bloom et al. (1956) divided the educational objectives into three domains: cognition, motor skills, and affectivity. Among them, the affective domain involves the change of emotion, value, appreciation, interest, motivation, or attitude that may be caused by learning experience, which emphasizes people's internal emotions and the degree of accepting or rejecting the things they pay attention to [1]. Russell (1986) cited Self-Concept and believed that interest, attitude, belief, value, self-esteem, morality, creativity, mental health, and self-development are all related to the affective domain [2]. Krathwohl et al.(1964) proposed the classification of goals in the affective domain, which can be divided into five levels from simple to complex: acceptance, response, value judgment, organization, and internalized value, indicating the degree to which affective characteristics are integrated into learners' behaviors [3].

In the face of major social and global challenges, the modernization of higher education must adhere to the core concept of being people-oriented, comprehensively improve the quality of higher education, and serve to promote the all-round development of people and sustainable social development [4]. Zhu XM believes that "whether at the level of human cognitive development, value cultivation, behavior learning, or at the level of deeper thinking structure, emotions play a holographic role as a "basic" existence" [5]. Medical educators not only undertake the responsibility of teaching skills and knowledge, but also shoulder the responsibility of cultivating medical students' morality, character, and humanism spirit and seek the best practice of cultivating doctors with all-round development [6–8]. The importance of the affective domain in training well-rounded doctors cannot be ignored. Learning in the affective domain (attitude, values and appreciation) is closely related to the development of specialty values [9]. Moreover, it is beneficial to cultivate medical students' humanistic spirit with medical ethics as the core and to cultivate medical students' specialized qualities such as empathy, affective care, and communication. To promote the all-round development of medical students, medical teachers must attach great importance to the affective domain in the medical application of specialty, fully tap the affective factors in the specialty, to guide medical students to form positive attitudes, beliefs and values unconsciously, deepening the medical students' understanding of specialized humanities connotation and the value, the medical humane spirit fosters into the whole process of teaching and clinical practice. The COVID-19 pandemic has affected the entire education system, especially medical education. With the shift to online teaching, helping medical students learn to value the emotion of others and become a competent doctor now requires teaching the affective realm more explicitly than ever before [10].

In recent years, Chinese medical education has increasingly focused on the affective domain. But the humanistic spirit of medical staff still can not meet the needs of patients' humanized care. Some studies show that the cultivation of a humanistic spirit of medical students is still focused on the pre-clinical basic teaching stage, In the stage of novitiate and practice, the teachers are mainly engaged in cultivating students' specialized skills, and it is difficult to take into account the cultivation of medical students' affective domain, resulting in the disconnection between theoretical knowledge and clinical practice, and it is difficult to achieve good cultivation effect [11]. We have previously conducted research from the perspective of students, using the student population as a sample. This research is based on the previous research, further expand the sample, and from the teacher's point of view, supplemented by student data, through the analysis of medical professional teachers' evaluation of their own affective field teaching, medical students' evaluation of teachers to comprehensively reflect the current situation and difficulties of medical teachers to carry out affective field teaching, in order to put forward a feasible path for medical teachers to implement affective education.

## Research design

### Ethics statement

We used a cross-sectional design for this research force. The Ethics Review Committee of Wenzhou Medical University approved the study (No. 2020–135).

### Participants

The participants were mainly teachers and students from medical schools in western China, including some of the teachers and students of medical universities. In this study, a random sample was conducted in the form of an online questionnaire for university teachers and undergraduates in China.

### Questionnaire

The overall structure design of teacher questionnaire and student questionnaire mainly refers to the four stages of Taylor's curriculum and teaching theory: determining educational objectives, selecting learning experience (teaching content), organizing learning experience and evaluating learning results. Specific indicators refer to FFT (Framework for Teaching, Danielson, 2013) based on constructivism Teaching theory [12]. It includes four modules: planning and preparation, classroom environment construction, classroom teaching skills, and specialized responsibilities; And the CLASS (Classroom Assessment Scoring System, Pianta, 2008) [13] includes modules of teacher affective support, classroom organization and teaching support, from which the common affective elements contained in teachers' teaching behaviors are summarized. The implementation effect of affective education in universities is not only related to the education and teaching behavior of individual teachers, but also to explore the reasons from the perspective of school management. According to the incentive theory, people's needs will produce motivation, motivation can stimulate behavior, pay attention to the mobilization of motivation to teachers' affective education enthusiasm, schools should establish a scientific and reasonable incentive mechanism, take corresponding incentive measures.

The Teacher Affective Education Implementation Scale for the Teacher Questionnaire consists of 20 questions and includes the following five dimensions: (1)Affective education attitude, mainly including teachers' care and respect for students, affective communication and other projects. Teachers' personal traits, knowledge, skills, and attitudes are needed to achieve effective teaching, and Tigelaar (2004) places special emphasis on focusing on teachers' positive attitudes toward students and how teachers perceive themselves as teachers [14]. (2) Affective education strategies, including affective education goals, instructional design, teaching methods and other projects; (3) Affective education evaluation is an indicator to measure the content, evaluation methods, and evaluation cycle of teachers' affective education evaluation of students. (4) Affective education cognition, including teachers' understanding of the connotation, value and basic knowledge of affective education and clarity about affective education goals. (5) School mechanism guarantees, including the evaluation mechanism, incentive mechanism, and role of school culture for teachers to carry out affective education.

The student questionnaire still uses the maturity scale from the previous small sample survey, which is composed of three parts: basic information, teacher affective education implementation scale, and student satisfaction scale for the effect of affective education, to understand students' views on teachers' affective education teaching behavior, and to self-evaluate the effect of affective education [15]. Due to the different roles of students and teachers in teaching, the affective education implementation scale in the student questionnaire removes the two dimensions of affective education cognition and school mechanism guarantee, and the affective education implementation scale of the student questionnaire consists of 14 questions. The Satisfaction Scale of Students' Effect of Affective Education consists of 16 questions, mainly referring to the five-layer classification model of affective domain goals of Krathwohl et al., which constructs a complete value internalization process for students to evaluate their own affective learning changes.

The Affective Education Implementation Scale for College Teachers and the Satisfaction Scale of Students with the Effect of Affective Education are scored by 5 points by Likert, and are rated from complete non-compliance to full compliance with 1–5 points.

### Data acquisition

We sent a questionnaire to all participants through a free online tool called "Wen Juan Xing" and told them that if they completed and submitted the questionnaire it would also be considered informed consent. In order to overcome the bias of the online questionnaire, we will remove the questionnaire that takes too long, too short or has logical errors before and after, and the survey collects a total of 467 (91.01%) valid teacher questionnaires and 2989 (91.46%) valid student questionnaires.

### Sample data description

In the data of teachers, 43.1% of them have been teaching for 0–5 years, 40.3% have been teaching for more than 10 years, and 16.6% have been teaching for 6–10 years. The samples of medical students were collected from three regions of eastern, western, and central China, among which the samples from eastern China accounted for 77%, and those from central and western China accounted for 38.9%. In terms of gender, males accounted for 31.7% and females 68.3%; In terms of grade distribution, freshmen and sophomores are the majority (59.1%), while juniors and sophomores accounting for 40.9%.

### Reliability test

**Fig. 1:**
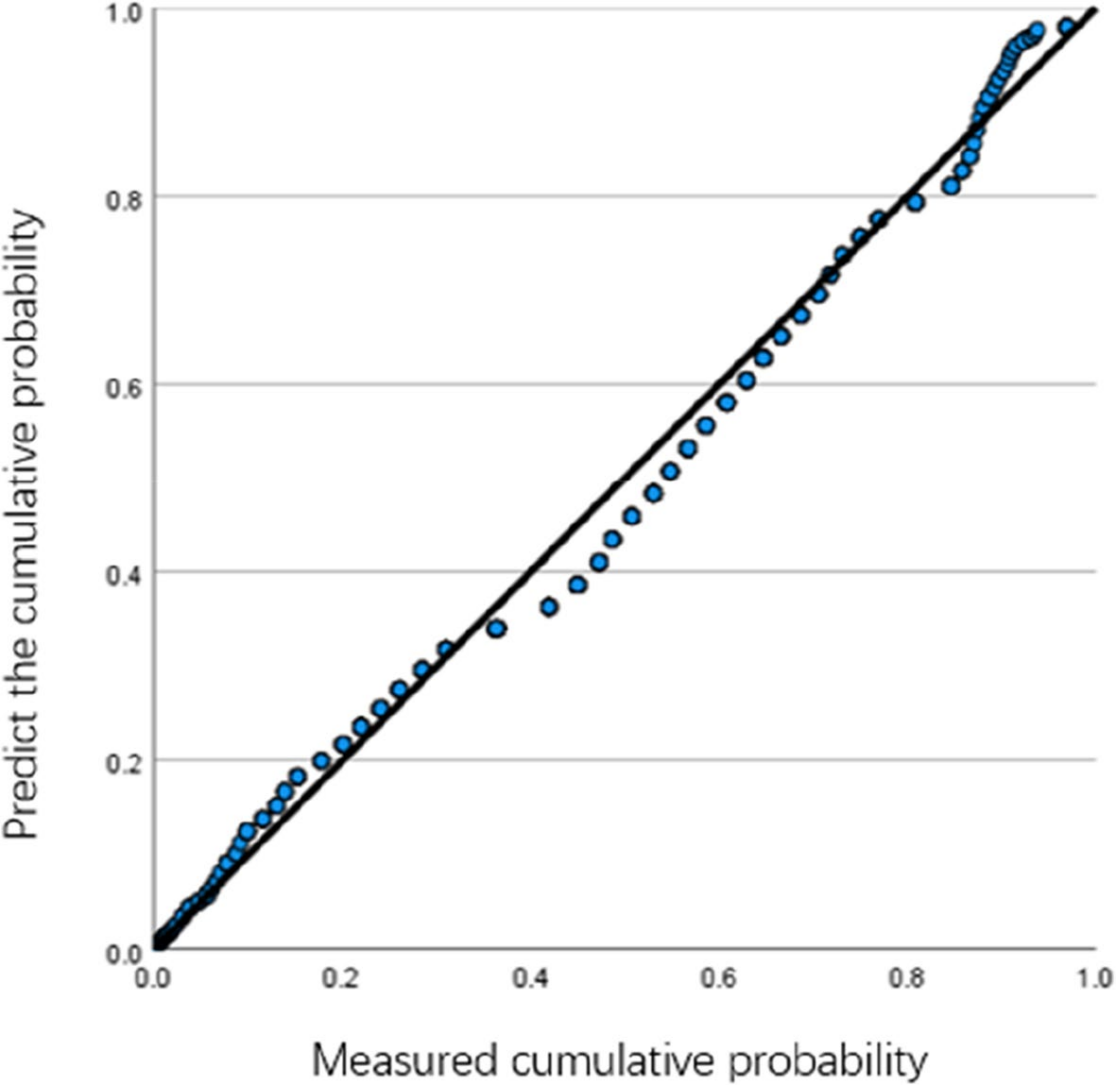
Teacher group

**Fig. 2:**
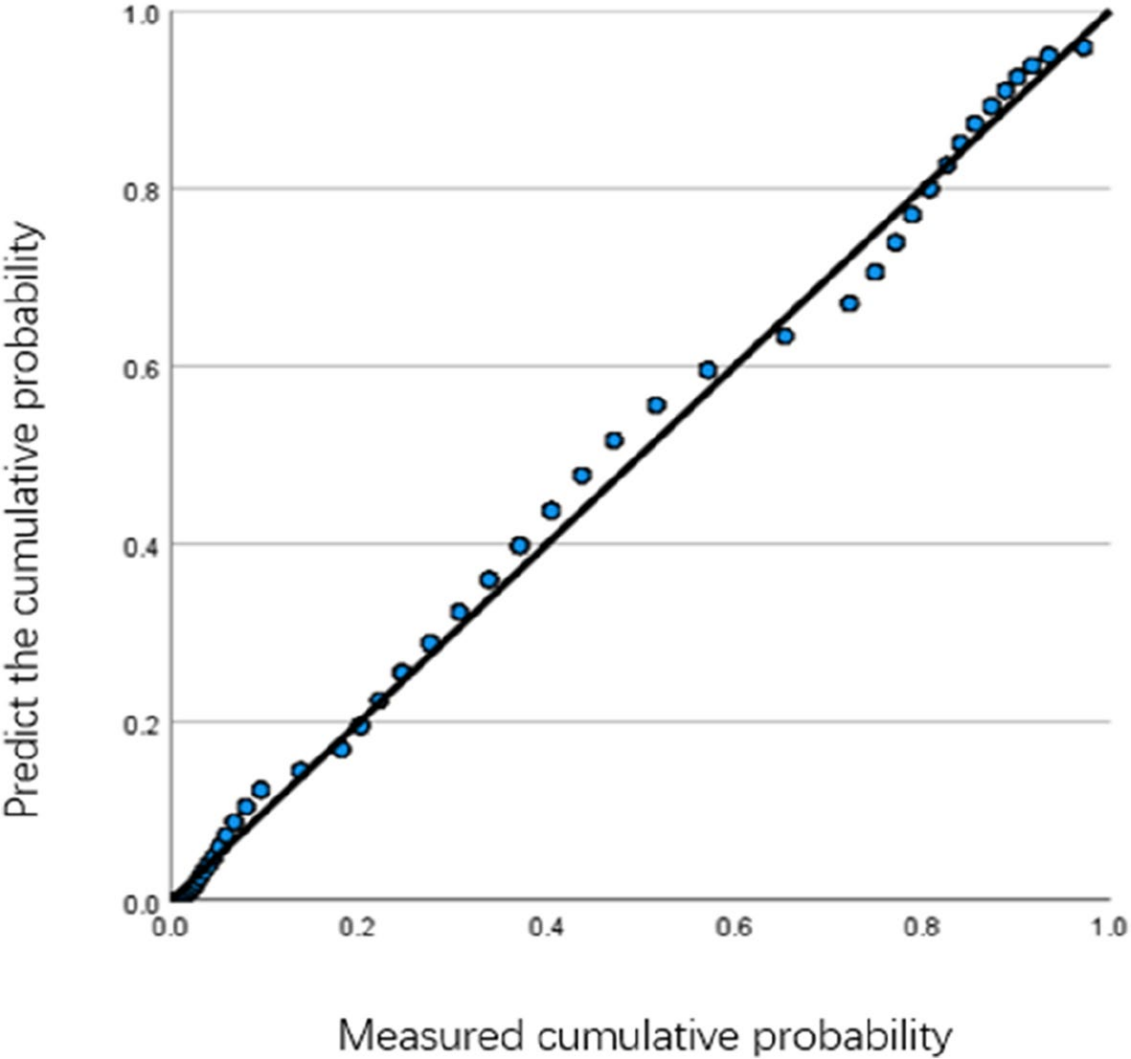
Student group

SPSS25.0 was used to conduct an internal consistency reliability test on the reclaimed questionnaire data. The Alpha coefficients of the teacher scale and student scale were 0.965 and 0.950, respectively, indicating good reliability of both scales. The results of the KMO and Bartlett sphericity test were 0.958 and 0.971 respectively, *p* < 0.001, indicating good data validity of the scale. Amos22.0 was used for confirmatory factor analysis of the scale, and the overall fitting coefficient was shown in Table 1. The χ^2^/df value of the teacher scale was 2.620, and that of the student scale was 2.201, both of which were lower than 3, in line with the model fitting standard, and the scale structure validity was good. SPSS25.0 was used to test the normal distribution of sample data for the affective education implementation scale of teachers and students, and the sample data shown in Figs. 1 and 2 were normally distributed.

**Table 1:** Overall fitting coefficient table

Indicators	χ^2^/df	RMSEA	NFI	RFI	IFI	TLI	CFI
Teachers (*N* = 467)	2.620	0.041	0.990	0.982	0.994	0.989	0.994
Students (*N* = 2989)	2.201	0.070	0.931	0.916	0.961	0.952	0.961

## Result analysis: The difficulties faced by medical university teachers in implementing affective education

### Unclear orientation and interpretation of affective education objectives

Because of the ambiguity and complexity of emotion itself, emotion-related teaching objectives are not as clearly defined as cognitive or action-related skills [16]. Affective goals describe the changes in interest, attitude and values, teachers tend to confuse affective goals with knowledge and skill goals, which makes it difficult to establish affective goals. According to teacher data, the mean value of "I am very clear about the affective education goal of the curriculum" was only 3.42, and 54% of teachers were still not clear about the affective education goal of the curriculum. Similarly, in the student data, the mean value of "most teachers have clearly proposed the affective education goal of the course" is significantly lower than the average level of the affective education strategy dimension (3.3 points), and the proportion of students who disagree with it reaches 56.%. From the perspective of both students and teachers, it is found that the ambiguous goal of teacher's affective education is a prominent problem. The reasons are as follows: first, teachers have a weak idea of integrating affective education into classroom teaching, not to mention taking actions with preset affective goals. From the perspective of the cognitive dimension of teachers' affective education, teachers' cognition of affective education is generally low, and 62% of teachers have a low understanding of the connotation and value of affective education; Second, although teachers realize the importance of affective education, they lack theoretical and practical learning of affective education, so they have a vague and general understanding of affective education and lack of sufficient ability to set affective goals. This is consistent with the cognitive results of affective education, 66% of teachers do not often read the literature on affective education, lack the background knowledge of affective education, and 70.2% of teachers have not received the training and guidance of affective education.

### Teaching strategies in the affective domain lack affective experience

The experiences that elicit affective responses in learners in health specialty education may occur in real patient care Settings or is designed simulated environments. This is conducive to promoting learning in the affective domain while obtaining the development of trainable skills in the psychomotor domain and knowledge in the cognitive domain. Table 2 items "I can use basic skills and methods of affective education in teaching practice" and Table 3 items "most teachers can provide opportunities for students to experience and practice" and the mean values are both low, indicating that some teachers lack basic skills of affective education. In terms of specific teaching methods, as shown in Table 4, the teaching methods that students have received the most are teaching method, discussion method, practice method, etc., while the teaching methods such as scenario simulation, role-playing, and affective communication have received the least; The top three teaching methods commonly used by teachers are lecturing, discussion and induction, and the three least frequently used are role-playing, cooperative learning, and affective communication. The results above show that some problems lack of affective education basic skills of teachers, most of the teaching is still in the teacher-centered knowledge stage, taking the student as the main body of the experience type teaching method is relatively small, teachers' lack of methods and strategies for implementation of affective education is a major obstacle to carry out the affective education in medical universities.

**Table 2:** Teacher scale

Dimension	Indicators	Means	SD
The attitude of affective education	I am good at self-emotion adjustment and management	3.60	0.927
	In teaching, I can find students' affective needs acutely and give timely understanding and response	3.63	0.908
	I am willing to guide students to solve problems in study and communication	3.78	0.912
Cognition of affective education	I understand affective education and understand its connotation and value	3.29	1.011
	I often read literature on affective education	3.14	1.060
	I have received formal training and instruction in affective education	2.90	1.192
Affective education strategy	I am very clear about the affective education goal of the course	3.42	1.014
	I often consciously integrate affective education into teaching design	3.49	0.961
	I can use the basic skills and methods of affective education in my teaching practice	3.36	0.964
	In my courses, I often discuss with students the humanistic connotation and value of major	3.46	1.022
	I can deeply interact with students, enlighten wisdom, edify sentiment	3.60	0.930
Evaluation of affective education	I can make a proper judgment and evaluation of the emotional changes of students in the course	3.53	0.916
	I often reflect on students' emotional changes and adjust teaching strategies in time	3.61	0.935
	I will evaluate the change in students' emotional and attitudes regularly	3.32	1.013
School mechanism guarantee	The school has formulated specific teaching guidelines for affective education	3.11	1.154
	The school has an organization specially responsible for the management of affective education	3.17	1.149
	The school has a complete evaluation system of teachers' affective teaching ability	3.06	1.131
	The school has teachers who can train affective education courses	3.05	1.143
	The school has a definite reward and punishment system for the result of affective education	2.98	1.146
	The school attaches great importance to the subjective status and initiative of teachers in affective education	3.22	1.091

**Table 3:** Student scale

Dimension	Item	Means	SD
Affective education attitude	Most teachers respect their students' individuality	4.00	0.865
	Most teachers can care for and protect the self-esteem and personality of underperforming students	3.93	0.875
	Most teachers are enthusiastic about teaching and keep a good emotional state	3.92	0.862
	Most teachers pay attention to their student's progress and give them timely recognition	3.87	0.910
	Most teachers can find students' affective needs in teaching and give timely understanding and response	3.71	0.937
Affective education strategy	Most teachers have proposed the affective education goal of the course	3.30	1.032
	Most teachers can deeply interact with students, enlighten wisdom and cultivate sentiment	3.81	0.903
	Most teachers often discuss the humanities and values of their major with their students in class	3.84	0.893
	Most teachers can integrate specialized historical backgrounds and events into teaching vividly and arouse students' affective resonance	3.89	0.855
	Most teachers provide opportunities for students to experience and practice	3.65	0.893
Evaluation of affective education	Most teachers will make appropriate judgment and evaluation of the affective changes of students in the course	3.66	0.927
	Most teachers encourage students to self-evaluate affectively	3.66	0.904
	Most teachers can provide positive, instructive, and supportive feedback to students	3.60	0.939
	Most teachers regularly evaluate their students' emotional and attitudinal changes	3.58	0.982

**Table 4:** Teaching methods

Item	Highest frequency of three (%)	Lowest frequency of three items (%)
The teaching methods that students have received	Teaching method (10.8%)	Scenario simulation (4.1%)
	Discussion method (9.8%)	Role-playing (3.7%)
	Practice method (8.3%)	Affective communication (2.8%)
Teaching methods used by teachers	Teaching method (11.6%)	Role-playing (3.2%)
	Discussion method (9.2%)	Cooperative learning (2.7%)
	Induction (8.5%)	Affective communication (2.6%)

### Lack of evaluation norms in the affective domain

As an analytical, formative and feedback evaluation, affective education evaluation means that teachers explain and judge students' affective changes, progress and achievements in the teaching process. The mean values of teachers' affective evaluation ranged from 3.32 to 3.61, and the mean values of students' evaluation of teachers' affective education ranged from 3.43 to 3.66. In addition, the lowest mean items in the data of teachers and students are both periodic evaluations, indicating that evaluation is a relatively weak link in the process of affective education. At present, teachers lack corresponding norms in the evaluation method, evaluation cycle, and evaluation content of affective education, especially the evaluation cycle. As for the question "how often do teachers evaluate students' affective domain?", the most frequent assessment was once a month (29.6%), while 12.8% said they never evaluate students' affective domain. On the one hand, the definition of the affective domain is broad and difficult to measure. Unlike motor skills or cognition, affective behaviors cannot be immediately seen and are difficult to assess [17]. On the other hand, it is difficult to be objective and fair in the evaluation of affective domain, and it is difficult to determine the evaluation criteria, which is easy cause controversy.

### Lack of continuity and progression in affective cultivation

Through Pearson correlation analysis, it was found that the three dimensions of teachers' affective attitude, strategy and evaluation were positively correlated with the results of students' affective education, and the results of the independent sample t test showed that the evaluation of the three dimensions of teachers' affective education by students of different grades was significantly different (t = 5.648 *p* < 0.01). The scores of first- and second-year students' affective attitudes, affective education strategies, and affective education evaluation dimensions were significantly higher than those of junior, senior, and fifth-year students, and their scores decreased with the improvement of grades. Taking the whole process of affective cultivation as the starting point to analyze its reasons. From the perspective of basic general education, its core concept is "whole-person education". However, the current general education courses in Chinese medical universities have such problems as "miscellaneous content, disorderly structure, poor quality and low status" [18]. Secondly, the cultivation of a medical humanistic spirit of medical students at present mainly relies on medical humanistic education courses, such as medical ethics, medical psychology, medical law, etc. Most of the courses are mainly theoretical teaching, and a large part of humanities and social science teachers in Chinese medical universities lack a systematic understanding of modern natural science and medicine [19]. These problems lead to the lack of profundity of general education and medical humanities education in the antecedent period of medical science and play a limited role in the affective cultivation of students in the later period. Thirdly, affective education is absent in later medical education. Most teachers of medical courses lack the idea of integrating affective education into the curriculum. Affective education, general education, medical humanities education, and specialized education need to form a progressive systematic system.

Then, we were interested in whether the factors that influenced the outcome of the two affective education were different, so we divided the student population sample into two groups, the lower grades (freshmen and sophomores) and the upper grades (the seniors, the fourths, and the fifths).and performed multiple regression respectively, and obtained two statistically significant regression models and the standardization coefficients of the respective variables, and the VIF was less than 5, indicating that there was no multicollinearity between the respective variables(Table 5).

**Table 5:** Regression models in the lower grades versus the upper grades

Dependent variable	Model 1(Lower grades)	Model 2(Upper grades)
Affective education attitude	0.174(0.025)	0.215(0.006)
Affective education strategy	0.142(0.031)	0.229(0.007)
Evaluation of affective education	0.555(0.023)	0.390(0.007)
Constant	0.667(0.057)	0.935(0.069)
R^2^	0.668	0.595
Correct the R^2^	0.667	0.594
F	1180.222	595.340
Sample size	1767	1222
*p* < 0.01		

The tables are all standardized coefficients, with standard errors in parentheses. The dependent variable is the mean of the student's affective education outcome

Comparing models 1 and 2, it can be seen that affective education attitudes, affective education strategies, and affective education evaluations explained 66.7% and 59.5% of the total variation of affective education results in lower and upper grade students, respectively. All three dimensions significantly positively affect the affective education outcomes of students of all grades, and the evaluation of affective education is the most influential factor among them, which is particularly prominent for lower grade students, which verifies our previous findings. However, the difference is that the influence of affective education attitudes on the affective education results of lower grade students is higher than that of affective education strategies, but in the upper grades, this situation is just the opposite, and the upper grades believe that affective education strategies are second only to the influencing factors of affective education evaluation. This tells us that after students enter the senior grades, the demand for affective education is gradually higher than the importance of affective education attitudes, and teachers should further improve the strategy of affective education for senior students.

### The status quo of affective education implementation of teachers of different teaching ages is different

Through the one-factor ANOVA test, the mean values of different teachers of different teaching ages in the implementation of affective education status quo in various dimensions were compared, and it was found that there were significant differences between different teaching ages in the dimensions of affective education cognition and school mechanism guarantee (*p* < 0.01, the variance is not uniform). The average affective education cognition and school mechanism guarantee of teachers with teaching years greater than 10 years are significantly lower than those of teachers with teaching years of 0–5 years and 6–10 years, respectively, while the difference between teachers with 0–5 years and 6–10 years of teaching experience is not statistically significant. This reminds us that teachers with longer teaching experience have lower affective education cognition than young teachers in long-term teaching work, and their satisfaction with the guarantee of school mechanisms is lower than that of young teachers. The reason for this may be due to the lack of motivation and ability of older teachers to learn in terms of affective teaching concepts and methods. China's university teacher teaching development center is mainly established in recent years, although the lack of teachers' affective education ability is a problem for teachers of all ages, but the center usually only focuses on the training of newly hired teachers and young teachers [20].

An empirical study about affective attitude and values goal preset and achieve pointed out that the teacher is the default affective goals and preset goals whether science, is the important embodiment of teachers' teaching design ability, it is not only related to teachers' teaching quality and sense of responsibility but also has a strong correlation with the school's teaching management and guidance [21]. School is an important carrier of affective education and a perfect organizational mechanism is an important guarantee for the practice of affective education. As can be seen from Table 2, the overall mean values of school organizational mechanisms are low, both below 3.5 points; and the item with the lowest mean value is " The school has a definite reward and punishment system for the result of affective education ". It shows that most schools lack a systematic and perfect organizational mechanisms of affective education, and need to improve the organizational structure of affective education, evaluation system of affective teaching ability, teaching reward and punishment mechanism, and teacher team construction. Medical universities should establish and perfect the organizational mechanism of affective education as soon as possible based on their characteristics and with the vision of sustainable development, to provide guidance and support for the integration of affective domain and medical specialty.

## Results discussion and implementation path exploration

### Schools should establish a sound organizational mechanism for affective education

It is found that there are some problems in the application of affective education in medical universities, such as the lack of clear goals of affective education, the lack of methods of affective education, and the lack of continuous and progressive implementation of effects of affective education, which are closely related to the lack of a perfect organizational mechanism in medical universities. At present, most medical universities do not have an organization responsible for the management of affective education, so schools can rely on professional departments such as the Teaching development Center to formulate the corresponding norms of affective education and provide all-round professional support and mechanism guarantee for teachers in the domain of affective goal setting, teaching design, teaching methods and evaluation standards of students' affective domain, to finally achieve the purpose of helping teachers to clarify their educational goals and improve their educational ability. Emphasis is placed on providing systematic affective skills training and guidance for teachers with a long teaching experience. On the one hand, it needs to provide systematic affective skills training and guidance for teachers. Formulate teaching guidelines for affective education, use the teacher development center to discover outstanding teachers, set up a professional team of teachers to offer affective education training courses; Regular organization of observation and teaching, simulation exercises, timely guidance and feedback. On the other hand, create a relaxed and harmonious cultural environment. Build an interactive learning platform for teachers, display and promote typical cases of different disciplinary backgrounds, fully respect teachers' subjective initiative, and provide a broader space for teachers. In addition, medical universities need to establish a sound incentive mechanism to encourage the minority of teachers who lack the willingness to carry out affective education, both must have the spirit and make the necessary material incentives to encourage teachers. Let them dare to innovate, to explore the affective education of personalized teaching mode.

### Improve teachers' affective quality from the inside out

Improving teachers' affective accomplishment in an all-round way is beneficial to the improvement of teachers' affective attitude, cognition, strategy, and evaluation. Different from teachers' ethics, affective literacy is closely related to teachers' personal experience, values, knowledge cultivation, and personality charm. From the point of view of the nature of "for teachers of teachers, affective accomplishment is the core of teacher's accomplishment as well as the unity of teacher's inner affective quality and externalized affective ability [22]. The inner affective quality should include moral character, humanistic accomplishment, ideals and beliefs, and educational spirit. Externalizing affective ability is manifested as affective expression, affective communication, and response in the interaction with students.

Firstly, the interdisciplinary perspective is used to systematically study the theory of affective education to deepen the teachers' understanding of the concept and value of affective education, enrich teachers' knowledge structure, broaden their educational horizon, and provide an endogenous impetus for the implementation of affective education. Secondly, schools should provide teachers with continuous practice domain of educational situation, in which teachers "take root" and "soak" [23], improve affective quality and exercise affective expression and communication ability in specific situations and problems. Thirdly, it is necessary to construct an "emotion - communication" type classroom and establish an affective teacher-student relationship. Through the smooth affective relationship between teachers and students in classroom teaching, the firm life connection, and the positive and healthy classroom teaching environment, the sound development of the whole personality including individual emotions can be finally achieved [24].

### Design clear progressive affective education goals and content system

Through interviews with teachers and experts, this study found that if we want to establish the goal of affective education, we need to answer the question first: whether students need to know the goal of affective education? Among them, some teachers and experts advocate that students should not be informed of affective education goals. They believe that many affective education goals are generated in the teaching process, and the results of students' affective education are the result of long-term cultivation, so students should be influenced subtly and get real feelings from experience. Conversely, we argue that students need to know specific and clear affective education goals at the beginning of the course, while avoiding narrowness and mechanization, and paying attention to capture the goals of affective education. Setting goals around appropriate attitudes and behaviors is considered crucial, for example, doctors should set clear expectations in their communication with patients and incorporate them into formative feedback and evaluation along with clinical knowledge and skills [25]. There is a need for consistency between the attitudes and values implicit in the educational curriculum and the characteristics of a specialty, and the best way to establish such consistency is for educators to disclose their expected learning outcomes and reward those who achieve those outcomes accordingly [26]. Based on establishing the common goals and beliefs of teachers and students, the experiential teaching strategy is adopted to make the affective education into the brain and heart through joint efforts of both sides and promote the sublimation of students' affective domain.

Teachers' curriculum design and training should pay attention to stratification and classification, according to the characteristics of students in different grades, excavate the teaching characteristics and rules of different subjects, and design definite progressive affective education goals and content systems that are integrated with specialized education. In terms of specialized knowledge, philosophical discussion, background allusion, contact with reality, experience sharing, etc., and giving full play to the positive role of affective elements, moisten things quietly guide students' value pursuit. According to the different content of affective goals, combining the needs of students' personal development and social needs, affective education goals can be established from the following three aspects: the first type is students' feelings towards themselves, such as self-respect and self-improvement; The second category is the affective goal related to specialty courses, that is, students' attitude and interest to the course itself, and their recognition of the humanistic connotation and value related to the major; The third category is the affective goals towards nature, society, and others, such as social responsibility and family and country feel, which have a positive impact on students' morality and values.

### Adopt experiential teaching methods

It is found that most teachers still focus on traditional teaching methods and lack experiential teaching methods and strategies, which tend to lead to the lack of profound affective education in medical universities. In the question "what are the teaching behaviors or teaching methods that are most helpful to your feelings, attitudes, and values?", students think the most helpful are teacher demeanor (14.1%), practical activities (11.9%), group cooperation (9.6%), scenario simulation (7.4%), role-playing (6.8%), communication and encouragement (6%). Students found the task-driven method (1.8%) and intuitive demonstration method (2.3%) to be the least helpful. Compared with the above results, it is found that the teaching methods that students are given the most are not consistent with the methods that help students the most with their affective attitudes and values. The most common method of instruction was only 4.4%, and student-centered teaching activities are more helpful to students' affective attitudes and values. Therefore, the teaching in affective domain should pay special attention to students' experience, adopt a multi-dimensional and deep reflective experience teaching strategy, emphasize practicality, subjectivity, interaction, and depth, and achieve the internalization effect of self-perception and self-inspiration by arousing students' affective resonance. For example, affective outcomes are sought and produced through teaching activities such as discussion, open debate, peer participation, role-playing, problem-based learning, participation in role models, simulations, games, group analysis of case studies, expert participation, and sharing ideas through reflection. Use strategies such as role modeling and mentoring in community-based medical education to provide opportunities for practice and integration with local organizations and communities to teach in the affective domain [27]. Muzyk et al. (2017) designed courses based on the framework of Bloom’s affective domain, using patient cases, interactive learning activities, and reflective discussion to increase pharmaceutical students' humanistic understanding of mental illness and appropriately change students' attitudes toward patients with mental illness [28].

### The construction of an affective education evaluation system

Traditional teaching evaluation mainly inspects students' mastery of knowledge and skills, which is mainly reflected in students' examination results. The final goal of affective education is to realize the all-round development of human beings, so the evaluation should return to the essence of emotion and examine whether students' cognition and behavior have positive changes in the implementation process of affective education.

Teaching evaluation has undergone three stages "measurement", "description" and "judgment". The first three generations of evaluation follow the scientism paradigm, while the fourth generation of evaluation theory adopts the constructivism paradigm, requiring the evaluation process to consider the needs of multiple value subjects [29]. Because the goals of affective education are mostly generative, the evaluation system of affective education should be considered from the perspective of the fourth generation of evaluation theory, in which formative evaluation runs through the whole process of teaching activities. Medical specialty’s affective evaluation subject should include students, teachers, government, schools, hospitals, patients, and to formulate appropriate affective education evaluation mechanism, in a specialty and curriculum, the classroom and clinical practice as the carrier, with humanity as the core of evaluation content, give priority to with formative assessment method. Through students' self-evaluation, teachers' evaluation, and classmates' evaluation, comprehensive description and judgment can be made, and finally, an evaluation model in the affective domain with students and teachers as the main stakeholders can be formed.

## Limitations

In this study, we investigate medical teachers and students in universities in the Middle East and western China, explore ways to improve the affective education of university teachers, and theoretically, the findings of this study can be generalized, but due toIn theory, the results of this study can be generalized, but due to the limitations of practical conditions, the sample collection data is limited to China, and the global scalability still needs to be further verified. This study finds differences between students of different grades and teachers of different teaching ages in various dimensions of affective education, but the more specific and detailed differences and the deeper reasons for this difference still need to be explored in future research.

## Conclusion

According to the survey results, teachers and students believe that the current teaching affective education goals in universities are not clear, the method of affective teaching is relatively single, the experiential teaching method is relatively few, the evaluation of affective education is particularly important for affective education but it is a relatively weak link in practice. This is consistent with our previous conclusions from a student's perspective and conducted a small-scale student sample survey. The study also found differences in affective education between students of different grades and teachers of different teaching ages, which will provide us with direction for improving medical affective education.

We recommend that universities improve the organizational mechanism of affective education, and provide guarantees and training according to the characteristics of teachers to achieve all-round improvement of their affective teaching literacy from the inside out. Moreover, according to the differences of students of different grades, we should design clear and progressive affective education goals and content systems, adopt more experiential teaching methods in teaching practice, build a scientific affective education evaluation system, and finally form an affective field evaluation model with students and teachers as the main stakeholders. This will not only shorten the psychological distance between teachers and students, but also improve the learning experience of medical students, which is conducive to the improvement of medical education level.

## Data Availability

The datasets used and analysed during the current study are available from the corresponding author on reasonable request.

## Abbreviations

FFT: Framework for Teaching
CLASS: Classroom Assessment Scoring System
KMO: Kaiser–Meyer–Olkin
VIF: Variance Inflation Factor
